# An Improved Method for Enhancing the Accuracy and Speed of Dynamic Object Detection Based on YOLOv8s

**DOI:** 10.3390/s25010085

**Published:** 2024-12-26

**Authors:** Zhiguo Liu, Enzheng Zhang, Qian Ding, Weijie Liao, Zixiang Wu

**Affiliations:** School of Information Science and Engineering, Zhejiang Sci-Tech University, Hangzhou 310018, China; liuzhiguo0404@126.com (Z.L.); fighting_dq_dz@163.com (Q.D.); 2023210703005@mails.zstu.edu.cn (W.L.); 2024220705043@mails.zstu.edu.cn (Z.W.)

**Keywords:** dynamic object detection, YOLOv8s, focused linear attention, GhostNet

## Abstract

Accurate detection and tracking of dynamic objects are critical for enabling skill demonstration and effective skill generalization in robotic skill learning and application scenarios. To further improve the detection accuracy and tracking speed of the YOLOv8s model in dynamic object tracking tasks, this paper proposes a method to enhance both detection precision and speed based on YOLOv8s architecture. Specifically, a Focused Linear Attention mechanism is introduced into the YOLOv8s backbone network to enhance dynamic object detection accuracy, while the Ghost module is incorporated into the neck network to improve the model’s tracking speed for dynamic objects. By mapping the motion of dynamic objects across frames, the proposed method achieves accurate trajectory tracking. This paper provides a detailed explanation of the improvements made to YOLOv8s for enhancing detection accuracy and speed in dynamic object detection tasks. Comparative experiments on the MS-COCO dataset and the custom dataset demonstrate that the proposed method has a clear advantage in terms of detection accuracy and processing speed. The dynamic object detection experiments further validate the effectiveness of the proposed method for detecting and tracking objects at different speeds. The proposed method offers a valuable reference for the field of dynamic object detection, providing actionable insights for applications such as robotic skill learning, generalization, and artificial intelligence-driven robotics.

## 1. Introduction

With the rapid advancement of robotics and artificial intelligence technologies, human-machine synergy [1] is becoming a critical direction for future development. To enable robots to perform tasks more efficiently, they must adapt flexibly to different environments and tasks, particularly in dynamic operational environments where effective object detection is crucial. In scenarios such as robotic assembly processes, accurate and efficient object detection plays a key role in enabling skill imitation [2] and learning. Compared to static object detection, dynamic object detection [3] demands higher accuracy and faster tracking of moving objects. Therefore, research on improving the accuracy and speed of dynamic object detection holds significant importance.

Traditional methods for dynamic object detection have been widely explored in both two-dimensional and three-dimensional contexts. Structured light projection 3D measuring techniques, including Fourier transformation profilometry (FTP) [4], phase-shifting profilometry (PSP) [5], and modulation measuring profilometry (MMP) [6], leverage precise optical measurements to capture object details in controlled environments. These methods are particularly effective in scenarios requiring high-precision measurements, such as industrial quality inspection. However, they are often constrained by environmental factors and require extensive calibration [7], limiting their applicability in dynamic and unstructured environments.

In contrast, 2D image processing methods, such as feature matching and correlation-based approaches, focus on analyzing pixel-level variations across frames to identify dynamic objects [8]. While computationally efficient, these methods rely heavily on handcrafted features and are sensitive to lighting conditions and noise, which restricts their effectiveness in real-world dynamic detection tasks. Among the 2D methods, frame differencing [9] and optical flow [10] analysis stand out for their application in dynamic environments. The frame difference method is to identify dynamic objects by comparing pixel differences between consecutive video frames. These methods compute the pixel-level difference [11] between the current frame and the previous frame to generate a differential image for dynamic object detection. The optical flow method analyzes pixel movements [12] across consecutive frames to infer the motion state of objects. By leveraging the differentiated characteristics of optical flow, these methods separate background motion from foreground objects, enabling dynamic object detection. However, both methods are sensitive to environmental conditions, such as illumination changes and occlusions, which limit their robustness in complex scenarios.

To address these limitations, modern methods leveraging deep learning [13] have emerged. The core idea of the deep learning method is to leverage convolutional neural networks (CNNs) [14] to automatically extract features from video frames and analyze spatiotemporal information for object recognition and localization. Temporal differencing method and optical flow method are highly sensitive to environmental conditions, whereas deep learning-based dynamic object detection approaches are capable of autonomously learning complex features from data [15], making them more effective in handling various visual patterns in dynamic object detection scenarios.

In dynamic object detection, the dynamic detection and tracking of assemblies is a long sequence task, affected by changes in the object’s position [16] and the angle of the collection device. As a result, the size of the objects and the background information also change. Due to the nature of dynamic detection, there is typically a clear separation [17] between the foreground and the background. Due to the dynamic nature of detection, which often involves a distinct separation between foreground and background elements, the training process can sometimes lead to model weight distribution issues. These challenges can potentially impact the effectiveness of the model in dynamic object detection.

To address these challenges, this paper proposes a method to enhance the detection accuracy and tracking speed of dynamic objects based on the YOLOv8s [18] architecture. The primary innovations of this method are as follows:

(1) The proposed method introduces a Focused Linear Attention mechanism into the YOLOv8s network to improve dynamic object detection accuracy; this module focuses on key features more effectively.
(2) Inspired by the Ghost module, the proposed method introduces Ghost convolution to replace the original convolution, reducing computational load. The model is further lightweighted on the basis of the optimized computational efficiency of the Focused Linear Attention mechanism.
(3) The proposed method uses VariFocal Loss as the classification loss function to optimize the network’s loss function, addressing the problem of imbalance between positive and negative samples caused by sample unevenness.

The remainder of this paper is organized as follows: Section 2 introduces related work on dynamic object detection. Section 3 presents the proposed method for improving the detection accuracy and speed of YOLOv8s for dynamic object detection. Section 4 provides a comprehensive explanation of the validation and comparative experiments for object detection. Section 5 discusses the research results and conclusions.

## 2. Related Work

There are various visual methods that have been proposed to achieve dynamic object detection tasks. These mainly include three methods: frame-difference-based methods, optical-flow-based methods, and deep learning-based methods.

### 2.1. Frame Difference-Based Methods

The frame difference method is a technique in dynamic object recognition that involves comparing consecutive frames of video to identify changes or movements, thus enabling the detection of dynamic objects. Yin et al. [19] proposed a dynamic difference learning method to model spatio-temporal inconsistencies by distinguishing inter-frame differences caused by facial manipulation from those caused by natural facial motions. Delibaşoğlu et al. [20] introduced a moving object detection method based on background modeling and subtraction. This tracking approach is used in background subtraction and simple frame difference to set weight during background subtraction operation. Zhang et al. [21] proposed a moving object detection algorithm combining the three-frame difference method and Online Moving Window Robust Principal Component Analysis (OMWRPCA). By using the OMWRPCA to extract the background image in the current frame and comparing it to the previous and current frames, the cavity and double shadow problems are avoided as well as the effects of background pixels.

### 2.2. Optical Flow-Based Methods

Optical flow-based methods analyze pixel movements across consecutive frames to infer the motion state of objects. By leveraging the differentiated characteristics of optical flow, these methods separate background motion from foreground objects, enabling dynamic object detection. Yang et al. [22] proposed an improved optical flow method using image pyramids, which first estimates optical flow coarsely on low-resolution images and then refines the estimation progressively on higher-resolution images, achieving robust motion tracking for dynamic objects. Hu et al. [23] developed a simultaneous localization and mapping algorithm for mobile robots based on dense optical flow. By calculating dense optical flow fields between adjacent frames and segmenting dynamic regions using adaptive thresholds, their approach enhances the accuracy of dynamic object relocalization. Ding et al. [24] propose a multiple-moving objects detection and recognition method that combines an optical flow histogram and K-means clustering analysis based on dense optical flow. The method first estimates the original optical flow field with the optical flow algorithm, then enhances the objects by a K-means algorithm, and finally filters out the noise with a self-adaptive threshold algorithm.

### 2.3. Deep Learning-Based Methods

The core idea of deep learning-based dynamic object detection methods is to leverage convolutional neural networks (CNNs) to automatically extract features from video frames and analyze spatiotemporal information for object recognition and localization. Tan et al. [25] proposed the EfficientDet algorithm, which systematically scales the network’s depth, width, and input resolution to improve detection speed at a low computational cost. However, its accuracy in complex scenes or for fast-moving objects remains insufficient. Carion et al. [26] introduced the DEtection TRansformer (DETR) algorithm based on a Transformer architecture. By combining CNNs and Transformers, DETR directly maps image features to object boxes and categories, enabling end-to-end detection. Redmon et al. [27] proposed the YOLO (You Only Look Once) model, which divides the input image into a grid and predicts a fixed number of bounding boxes and confidence scores for each grid. By aggregating the predictions from all grids, YOLO generates the final detection results. This fast detection model sparked a wave of research in machine vision applications. Wang et al. [28] developed the YOLOv7 model based on the YOLO architecture, which integrates features from multiple scales using a feature pyramid network and adaptive feature fusion. While this approach improves detection accuracy, it results in reduced detection speed. Ultralytics [18] further proposed the YOLOv8 series models, which incorporate CSPNet to optimize the network structure by splitting and merging feature maps through Bottleneck modules. These Bottleneck modules enhance feature extraction by compressing and expanding feature channels, effectively improving the model’s ability to capture diverse representations while reducing computational cost. These enhancements aim to balance detection accuracy and speed. Kang et al. [29] developed the novel BGF-YOLO architecture, which incorporates an attention mechanism to focus on critical features. Additionally, it employs a feature pyramid network to merge high-level semantic features with spatial details, enriching the feature representations and improving detection accuracy. An et al. [30] developed a dynamic convolution YOLO fire detection method, which uses the K-means++ algorithm to optimize anchor box clustering and introduce the dynamic convolution into the convolution layer. Zhang et al. [31] combined the YOLO model with a background subtraction algorithm for the dynamic detection of rolling rocks. They addressed overfitting by adopting activation functions tailored to specific scenarios and integrated a Gaussian mixture model-based background subtraction algorithm to accurately identify moving rocks on railway slopes. Zhao et al. [32] proposed a novel real-time object detection system that incorporates the convolutional block attention module (CBAM) and a self-attention mechanism to enhance the YOLOv8s model, designed to address the challenges posed by indistinct bottom boundaries and foggy imagery.

In summary, the three categories of dynamic object detection methods exhibit distinct characteristics. The first category identifies moving objects by calculating pixel differences between consecutive frames. While simple and computationally efficient, these methods are sensitive to illumination changes and prone to noise interference. The second category captures object motion by calculating motion vectors of pixels in image sequences, enabling the detection of subtle motions and continuous trajectories. However, these methods involve high computational complexity and are significantly affected by illumination changes and noise. The third category, exemplified by the YOLO series, can automatically extract image features and offers strong accuracy and real-time performance, making it advantageous for dynamic object detection scenarios. Nevertheless, there remains room for further improvement in the trade-off between detection speed and accuracy.

## 3. Proposed Method

**Figure 1:**
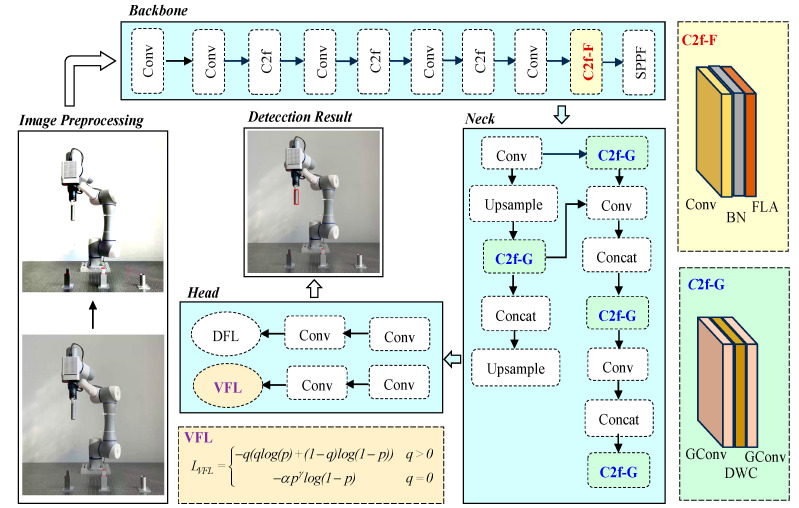
Dynamic object detection method based on the improved YOLOv8s architecture. The light blue modules represent the original YOLOv8s architecture (Backbone, Neck, and Head). The light yellow module (C2f-F) enhances feature representation in the Backbone using Focused Linear Attention. The light green module (C2f-G) optimizes multi-scale feature fusion in the Neck using Ghost Convolution. The brown–yellow module (VFL) addresses the class imbalance in the Head by introducing VariFocal Loss.

The proposed method is based on the YOLOv8s architecture, which consists of three primary components: Backbone, Neck, and Head. As shown in Figure 1, the light blue regions correspond to the original YOLOv8s modules, which serve as the foundational framework for our improvements. The Backbone extracts multi-level features through convolutional layers, the Neck fuses multi-scale feature maps, and the Head predicts bounding boxes and class probabilities.

The Backbone of YOLOv8s, as represented in light blue, is responsible for multi-level feature extraction. To enhance its ability to capture critical features, the proposed method integrates a C2f-F module (highlighted in light yellow), which incorporates a Focused Linear Attention (FLA) mechanism. This addition strengthens the representation of key regions in dynamic object detection tasks. The Neck of YOLOv8s (light blue) integrates feature information from different hierarchical levels. To further optimize this process, the proposed method replaces standard convolutions with the C2f-G module (light green), which leverages Ghost Convolution to reduce computational costs while maintaining feature quality. Moreover, the Head module (light blue) in YOLOv8s generates predictions for bounding boxes and class probabilities. To address the class imbalance in dynamic object detection tasks, the proposed method integrates a VariFocal Loss (VFL) module (highlighted in brown–yellow), which adjusts the weights of positive and negative samples during training.

While the original YOLOv8s provides a robust framework for real-time object detection, its standard convolutional and loss mechanisms show limitations in handling dynamic object detection scenarios. Specifically, Backbone’s standard convolutions lack focus on critical dynamic features, which is addressed by the proposed C2f-F module. The Neck’s conventional fusion method involves high computational costs, mitigated by the C2f-G module. The Head’s BCE loss is insufficient to balance positive and negative samples in dynamic environments, but it has improved through the introduction of VFL.

### 3.1. Improved YOLOv8s Backbone with C2f-F

**Figure 2:**
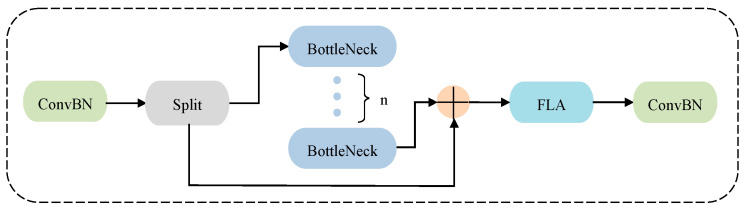
Structure of the C2f-F module.

In the YOLO architecture, the Backbone is responsible for extracting features from the input image, reducing the size of feature maps, and enhancing semantic information through convolution and pooling operations. In this study, a Focused Linear Feature Learning module, named C2f-F, is proposed for the Backbone, shown in Figure 2. This module is strategically placed at the end of the Backbone to extract and enhance the feature representation of the input image via convolutional and pooling layers. By inserting Focused Linear Attention before the final convolution of the original module, C2f-F enhances the ability to capture critical features while reducing computational complexity.

In the C2f-F module, the input feature map is denoted by *X*∈*R^c^*^×*h*×*w*^, where *c* represents the number of channels, and *h* and *w* denote the height and width of the feature map, respectively. As shown in Equation (1), *F_in_* represents the initial features extracted through convolution (*Conv*) and batch normalization (*BN*).

(1) $$F_{in}=ConvBN(X)$$

where *F_in_* represents the feature map after initial convolution processing, *X* denotes the input feature map, which comes from the output of the previous layer, and *ConvBN* refers to the convolution and batch normalization operations.

Afterward, the feature map is divided into multiple branches to obtain *F_split_*, allowing for parallel processing in the subsequent Bottleneck modules. Then, Equation (2) is used to fuse the features from multiple Bottleneck modules, resulting in *F_bottlenck_*.

(2) $$F_{bottleneck}=\sum_{i=1}^{N}Bottleneck(F_{split})$$

where *N* represents the number of Bottleneck modules, and *Bottleneck*(*F_split_*) denotes the feature map after each split branch *F_split_* is processed by the Bottleneck module.

Feature extraction is performed on the fused feature map through convolutional operations, as shown in Equation (3).

(3) $$Q=Conv_{Q}(F_{bottleneck}),\;K=Conv_{K}(F_{bottleneck}),\;V=Conv_{V}(F_{bottleneck})$$

where *Q*, *K*, and *V* represent the query matrix, key matrix, and value matrix, respectively, and Conv denotes the convolution operation used to compute the queries, keys, and values. After this, FLA is applied for attention enhancement.

**Figure 3:**
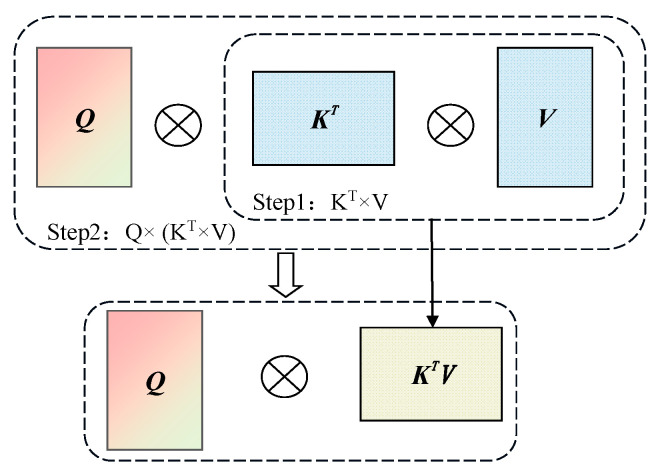
Structure diagram of linear attention mechanism. The linear attention mechanism first computes the aggregation of values by multiplying the transposed key matrix *K^T^* and the value matrix *V*. The result is then multiplied with the query matrix *Q* to generate the final output.

As shown in Figure 3, the linear attention mechanism operates by first aggregating values through the multiplication of the key matrix *K^T^* and the value matrix *V*. The result is then multiplied with the query matrix *Q* to generate the final output. This mechanism reduces the computational complexity compared to traditional attention mechanisms by avoiding the computation of the full attention matrix *Q* × *K^T^* and replacing softmax operations with linear transformations. However, it suffers from limited expressiveness in capturing complex feature interactions due to its linear nature, which can lead to suboptimal performance in dynamic object detection tasks. FLA redesigns the attention calculation method based on linear attention, proposing a focused mapping function *f_p_* to address the issue of output averaging in linear attention. The specific formula can be expressed as:

(4) $$f_{p}(x)=\frac{∥x∥}{∥x^{**p}∥}x^{**p}$$

where *f_p_* represents the mapping function, *x* denotes the elements in the function, and x^**p^ indicates the element-wise power *p* of *x*.

The mapping function *f_p_* adjusts the feature direction by multiplying ‖*x*‖ with x^**p^, followed by a ratio operation with ‖x^**p^‖. During the operation, the feature norm remains unchanged; only the direction of the features is modified. This function aligns similar items closer and separates dissimilar ones by adjusting the directions of queries and keys, enhancing the linear attention mechanism’s ability to extract key features and improving the model’s detection accuracy.

Additionally, DepthWise Convolution (DWC) is introduced in FLA to enhance the rank of the value matrix within the attention mechanism, further improving feature representation capabilities. By incorporating DWC operations into the attention matrix, the attention computation complexity is reduced from *O*(*n*^2^) to *O*(*n*), as compared to traditional attention mechanisms. This process combines the focused mapping function and DWC operations, as detailed in Equation (5):

(5) $$O=Sim(Q,K)V=f_{p}(Q)f_{p}{\left(K\right)}^{T}V+DWC(V)=f_{p}(Q)f_{p}{\left(K\right)}^{T}V+DWC(V)$$

where *f_p_* represents the focused mapping function, *Q*, *K*, and *V* denote the query, key, and value matrices, respectively, *T* indicates transposition, *DWC* represents the DepthWise Convolution operation, and *O* is the output feature.

By adjusting the directions of the query *Q* and key *K* features through *f_p_*(*Q*)*f_p_*(*K*)*^T^*, the attention weights among features are effectively computed. The *DWC* operation enhances the diversity and rank of the value matrix *V*, improving feature representation. The enhanced feature maps are then processed through convolutional layers to produce the output.

The FLA mechanism enables attention enhancement for both global and local information in the feature map, allowing the C2f-F module to more precisely capture key features. This prevents detection inaccuracies caused by dispersed feature weights and significantly improves the model’s understanding and representation of deep image features. Consequently, it enhances detection accuracy effectively.

### 3.2. Improved YOLOv8s Neck with C2f-G

**Figure 4:**
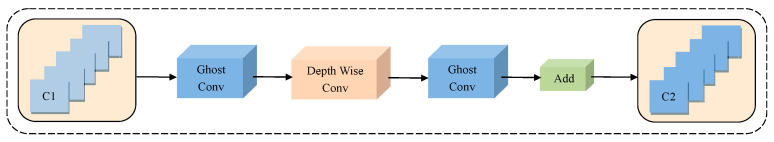
Structure of the Ghost Bottleneck.

In the YOLO network, the Neck layer effectively integrates feature information from different hierarchical levels through upsampling and feature fusion operations, enhancing the expressiveness of the features. By adjusting the scale, the Neck layer generates feature maps of different sizes to accommodate the detection of objects of various dimensions. In this study, the Neck layer is optimized using the principles of GhostNet, introducing the Ghost Bottleneck structure. The Ghost Bottleneck layer replaces the conventional Bottleneck layer in the C2f module by incorporating Ghost modules to improve feature processing efficiency. Using parallel branches, the Ghost Bottleneck reduces computational complexity while enhancing the model’s detection speed. The structure of the Ghost Bottleneck is shown in Figure 4. This modification reduces computational load while enhancing feature expressiveness by retaining important high-level features through linear transformations. By using a reduced number of feature maps and generating additional features through efficient linear operations, the model’s ability to process multi-scale features is improved, which accelerates inference speed.

This structure consists of two 1 × 1 convolutional layers. The first layer expands the channel dimensions, and the second layer restores them, maintaining consistency with the input channel dimensions. DepthWise Convolution is introduced to perform downsampling and feature restoration. Furthermore, all conventional convolutions in the Ghost module are replaced by PointWise Convolution, further reducing computational complexity and gradient propagation overhead. Through these efficient convolutional operations, the Ghost Bottleneck structure minimizes gradients and decreases computational complexity, thereby significantly improving the model’s detection speed.

In the Ghost Bottleneck layer, given input data *X*∈*R^c^*^×*h*×*w*^, where *c* is the number of input channels, and *h* and *w* are the height and width of the input data, respectively, the operation of generating *n* feature maps in any convolutional layer can be expressed as:

(6) Y=X*f+b

where *Y* represents the output feature map, *f* denotes the convolution kernel, * indicates the convolution operation, and *b* is the bias term.

**Figure 5:**
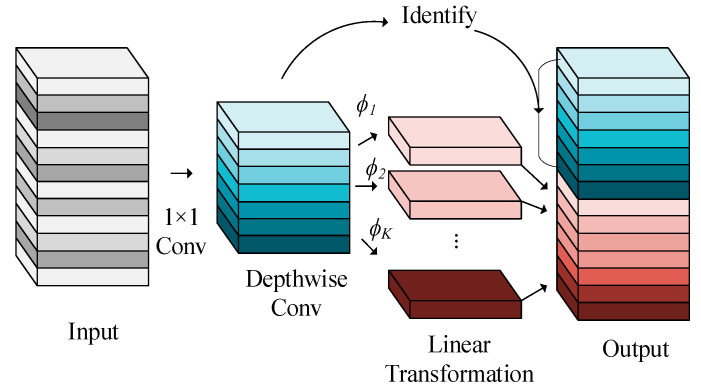
Structure of the Ghost Module.

The schematic diagram of the Ghost Module is shown in Figure 5. The Ghost Module first employs a 1 × 1 convolution to integrate features and generate initial feature maps, followed by DepthWise Convolution for further processing to produce Ghost feature maps. This process is followed by a linear transformation, where each feature map undergoes a lightweight linear operation to generate multiple Ghost features. This linear transformation reduces the computational cost by generating a large number of features from a single feature map. By doing so, it effectively extracts rich feature information while maintaining high computational efficiency. This transformation allows the model to capture diverse feature representations without increasing the number of convolutions or requiring substantial computational resources, thus improving both feature expressiveness and processing speed.

Through this lightweight operation, the Ghost Module generates a small number of feature maps using conventional convolution kernels, and the remaining feature maps are generated via inexpensive linear transformations. This process can be expressed as:

(7) $$Y^{\prime }=X*f^{\prime }$$

where *Y*′∈*R^h^*′^×^*^w^*′^×^*^m^* represents the output features, with *h*′, *w*′, and *m* denoting the height, width, and number of output channels, respectively, and *f*′∈*R^c^*^×*k*×*k*×*n*^ representing the convolution kernel.

The number of channels *m* in the output feature maps is less than the *n* value of a conventional convolutional layer, and the bias term is neglected to simplify the model. To further generate *n* mappings, a cheap linear operation is performed on *Y*′, generating *s* Ghost features through a feature map function:

(8) $$Y_{ij}=\phi_{ij}({y_{i}}^{\prime }),\forall i=1,\ldots ,m,j=1,\ldots ,s$$

where *ϕ_ij_* represents the linear transformation, *y_i_*′ is the intrinsic feature map of *Y*′, *m* denotes the number of channels, and *s* represents the number of features.

The intrinsic feature mappings *y_i_*′ generate Ghost feature mappings through lightweight linear transformations *ϕ_ij_*. Each feature map produces *s* Ghost features through these transformations, resulting in a final computational cost of *m* × *s* × *h*′ × *h*′ × *k* × *k*. As shown in Formula (7), the initial feature maps are processed using a simple 1 × 1 convolution, followed by Depthwise Convolution to extract low-level features. The resulting feature maps, represented as *Y*′, are then subjected to a linear transformation, as expressed in Formula (8), where each feature map produces *s* Ghost features. This approach reduces the number of convolutional operations required while preserving the feature diversity needed for accurate object detection.

The lightweight nature and efficient feature generation of the Ghost Bottleneck enable the C2f-G module to generate multi-scale feature maps effectively. This structure enables the YOLO network to process features rapidly, accelerating inference speed and improving detection efficiency. Through this design, the proposed method achieves effective multi-scale feature fusion in the Neck layer, enhancing inference speed and resource efficiency without compromising detection accuracy.

### 3.3. Loss Function

In the YOLO network, the Head layer applies convolutional operations on each feature map scale to predict the final detection results. These predictions are then fed into the loss function, where the losses are weighted and summed to compute the final loss value. In the proposed method, the VariFocal Loss (VFL) is adopted to replace the traditional Binary Cross Entropy With Logits Loss (BCEWithLogits Loss) to address the issue of class imbalance between positive and negative samples. VFL combines Binary Cross Entropy Loss (BCE Loss) and Focal Loss, and its formulation is expressed in Equation (9):

(9) $$L_{VFL}=\left\{\begin{array}{cc} -q(qlog(p)+(1-q)log(1-p)) & q>0\\ -\alpha p^{\gamma }log(1-p) & q=0 \end{array}\right.$$

where *p* represents the model’s predicted classification probability, *α* and *γ* are the modulating parameters of Focal Loss, and *q* is the ground truth value. For positive samples, *q* ranges from 0 to 1; for negative samples, *q* = 0. In the loss computation process, when *q* > 0, BCE Loss is employed to address the weighting of positive samples. When *q* = 0, Focal Loss is applied to balance negative samples, mitigating the impact of the class imbalance between positive and negative samples.

Traditional BCEWithLogits Loss does not differentiate between the weighting of positive and negative samples. In dynamic object detection tasks, the imbalance between positive and negative samples is particularly pronounced due to the large proportion of background regions. The introduction of VFL dynamically adjusts the weights of positive and negative samples, improving the model’s detection performance. By employing the VFL loss function, the proposed method better addresses the continuity of background regions in dynamic object detection. This effectively enhances the model’s detection accuracy and robustness.

### 3.4. Experimental Study

#### 3.4.1. Datasets

To evaluate the detection capabilities of the proposed algorithm in laboratory scenarios, a custom dataset was created. This dataset includes six different categories: cylindrical assembly front view, cylindrical assembly side view, cuboid assembly front view, cuboid assembly side view, triangular prism assembly front view, and triangular prism assembly side view. Using Azure Kinect DK and RealSense cameras, 2935 images were captured from various angles of the experimental setup under different background and lighting conditions.

In the raw images, the side view of cylindrical assemblies consistently appears as rectangles. In contrast, the side views of cuboid and triangular prism assemblies exhibit varied shapes due to their edge characteristics under different angles. To mitigate the issue of class imbalance in the dataset, a foreground segmentation-based method was adopted. Representative side-view images of the assemblies were first cropped from the original images, and their pure pixels were extracted. Subsequently, these side-view pixels were combined with background images to generate new training images. Data augmentation techniques were applied to the resulting images, ultimately producing a dataset containing 7234 images. This dataset was divided into training, validation, and test sets in a 7:1.5:1.5 ratio. Details of the dataset composition are presented in Table 1.

In addition, to validate the robustness of the algorithm, the publicly available MS-COCO dataset was utilized in this study. MS-COCO contains 80 object categories, with 118,000 images in the training set for training object detection models and 5000 images in the validation set for model validation.

#### 3.4.2. Experimental Setup

**Figure 6:**
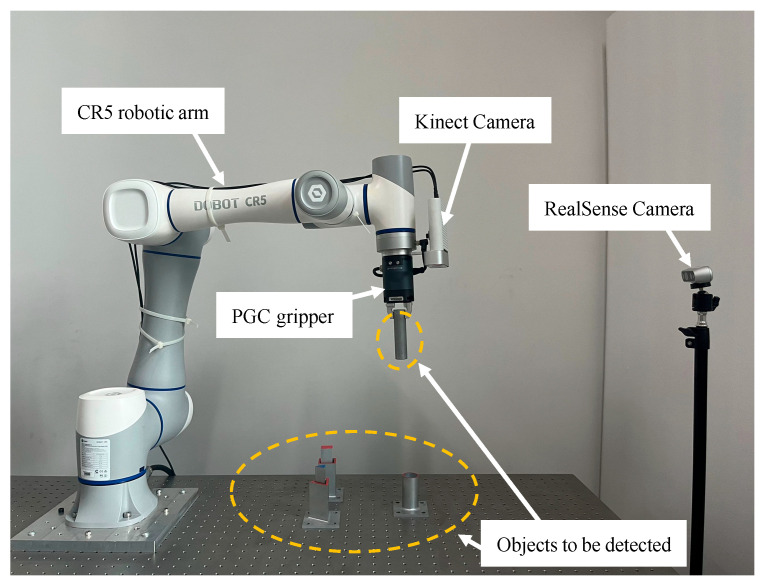
Experimental Setup.

To verify the feasibility of the proposed method for dynamic object detection tasks, a hardware experimental setup was constructed, and related experiments were conducted. The hardware setup for the dynamic object detection task is shown in Figure 6. The main components include the DOBOT CR5 robotic arm, Kinect camera, PGC-50-35 gripper, RealSense camera, and the assembling objects to be detected.

The model’s training and testing tasks were performed on a system configured as specified in Table 2. The training parameters were set as follows: Input image size: 640 × 640, total training epochs: 100, batch size: 16, Intersection over Union (IoU) threshold: 0.5. These parameters collectively determine the training process and the criteria for evaluating the model’s performance.

#### 3.4.3. Evaluation Metrics

In this study, the basic evaluation metrics for detection accuracy include Precision, Recall, and mAP (mean Average Precision), defined as follows:

(10) $$Precision=\frac{TP}{TP+FP}$$

where *TP* (True Positive) represents the number of samples correctly predicted as positive by the model, and *FP* (False Positive) represents the number of samples incorrectly predicted as positive. Precision measures the proportion of correctly predicted positive samples among all positive predictions.

(11) $$Recall=\frac{TP}{TP+FN}$$

where *FN* (False Negative) represents the number of samples incorrectly predicted as negative. *Recall* measures the proportion of actual positive samples correctly predicted by the model.

(12) $$mAP=\frac{\sum_{1}^{k}\int_{0}^{1}Percision(Recall)d(Recall)}{N}$$

where *mAP* denotes the average precision score, *N* is the total number of classes, and *k* ranges from 0 to *N* − 1. mAP serves as a comprehensive performance indicator for object detection models across various thresholds.

To evaluate the real-time performance of dynamic object detection, FPS (frames per second) and Inferenced Time are used as performance metrics:

(13) $$FPS=\frac{Number\;of\;Frames\;Processed}{Total\;Processing\;Time\;(seconds)}$$

where *FPS* measures the inference speed of the model.

(14) $$Inference\;Time\;=\frac{1}{FPS}*1000$$

where *Inference Time* measures the inference time in milliseconds for the model. Additionally, GFLOPs and parameters are employed to evaluate the computational complexity of the model.

## 4. Experimental Results

### 4.1. Ablation Study

To evaluate the effectiveness of the optimization strategies for each module in the proposed method—including the C2f-F module, C2f-G module, and VFL—and the impact of different module combinations on model performance, ablation experiments were conducted on the custom dataset. The experimental results are summarized in Table 3.

The experimental results indicate the following: When the C2f-G module was introduced individually, detection speed improved significantly compared to the baseline model, with FPS increasing by 19.7, parameters reduced by 2.8 M, and inference time reduced by 0.8 ms. This demonstrates the module’s effectiveness in optimizing detection speed. When the C2f-F module was introduced, the mAP@0.5 improved by 3.2%, validating its capability to enhance feature extraction and improve detection accuracy. Replacing the classification loss function with VFL increased the mAP@0.5 by 2.1%, showing its advantage in addressing class imbalance and boosting detection accuracy. When combining the C2f-G and C2f-F modules, the model achieved a balance between accuracy and speed, with the parameter count reduced to 9.4 M. When all three modules were integrated, the model achieved the best detection performance, with a mAP@0.5 of 92.6%, an improvement of 4.1% over the baseline model, an FPS of 166.9, GFLOPs of 24.6, an inference time of 5.9 ms, and a parameter count of 9.4 M.

These results confirm the effectiveness of the proposed method: The C2f-F module significantly enhances detection speed. The C2f-G module and VFL improve detection accuracy. Under the combined effect of these three modules, the proposed method achieves optimal detection performance. Therefore, the improved YOLOv8s model with these integrated optimization strategies will be used in subsequent experiments.

### 4.2. Comparative Experiments on Public Datasets

**Figure 7:**
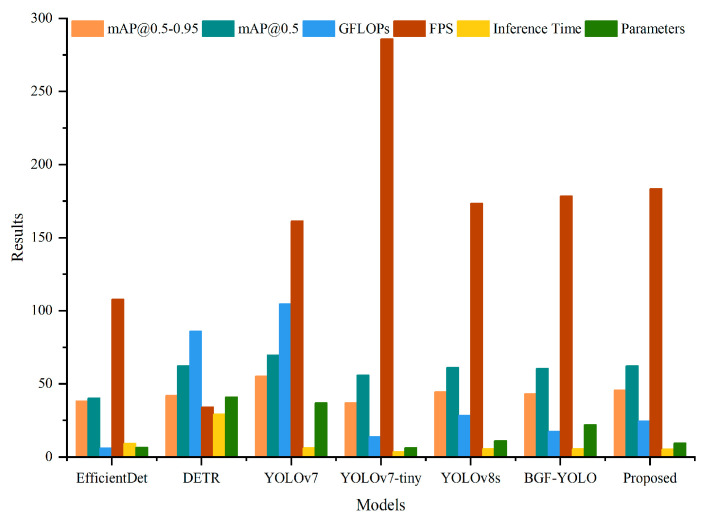
Results of object detection comparison experiments on the COCO dataset.

To validate the performance of the proposed method on public datasets, comparative experiments were conducted under the same experimental conditions. The proposed method was compared with six state-of-the-art object detection methods: EfficientDet, DETR, YOLOv7, YOLOv7-tiny, YOLOv8s, and BGF-YOLO. The experiments were performed on the MS-COCO dataset, and the evaluation metrics included mAP@0.5–0.95, mAP@0.5, GFLOPs, and parameter count. The comparative results on the public dataset are presented in Table 4 and Figure 7.

The experimental results demonstrate the following: In terms of detection accuracy, the proposed method achieved a mAP@0.5–0.95 of 45.3% and a mAP@0.5 of 62.2%, which are improvements of 0.7% and 1%, respectively, compared to YOLOv8s. Among the seven methods, the proposed method ranked second in accuracy, surpassed only by YOLOv7. Meanwhile, in terms of detection speed, the proposed method achieved a GFLOPs value of 24.6, significantly reducing computational complexity compared to GFLOPs-heavy methods like DETR and YOLOv7. While YOLOv8s and BGF-YOLO recorded GFLOPs of 28.4 and 17.4, respectively, the proposed method demonstrated a good balance of efficiency. The proposed method’s parameter count was 9.4 M, making it the third lightest model among all compared methods, following EfficientDet and YOLOv7-tiny. Compared to YOLOv7 and DETR, the proposed method achieved an effectively reduced parameter count, rendering it more lightweight.

The experimental results indicate that the proposed method strikes a good balance between accuracy and computational resource consumption on the COCO dataset. Despite having lower GFLOPs and fewer parameters, the proposed method achieved high detection accuracy, demonstrating competitive overall performance.

### 4.3. Comparative Experiments on the Custom Dataset

**Figure 8:**
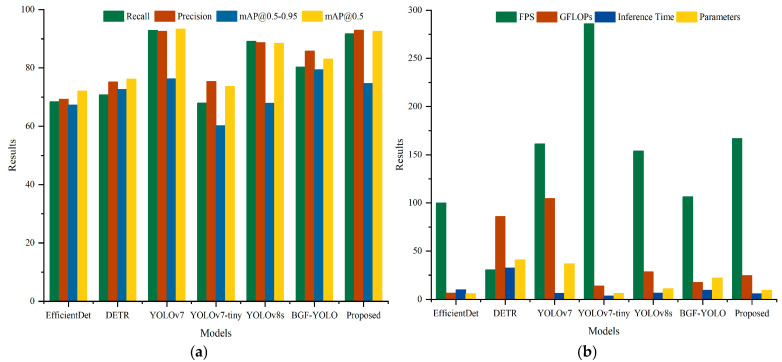
Results of object detection comparison on the custom dataset. (**a**) Object detection accuracy results. (**b**) Object detection speed results.

To evaluate the effectiveness of the proposed method in improving detection accuracy and speed on the custom dataset, comparative experiments were conducted under identical conditions. The proposed method was compared with six state-of-the-art object detection methods. The comparative results are presented in Table 5 and Figure 8.

The experimental results show that in terms of detection accuracy, the proposed method achieved a Precision of 93.0%, the highest among all compared methods. The mAP@0.5 reached 92.6%, ranking second only to the high-parameter YOLOv7. Additionally, the Recall and mAP@0.5–0.95 were 91.7% and 74.7%, respectively, demonstrating the method’s robustness while maintaining high accuracy. In terms of detection speed, the proposed method achieved an FPS of 166.9, second only to YOLOv7-tiny, while maintaining a low parameter count of 9.4 M and an inference time of 5.9 ms. This result indicates that the proposed method balances high accuracy and fast detection effectively.

The experimental results confirm that the proposed method performs well in both detection accuracy and speed on the custom dataset.

To provide a more intuitive demonstration of the experimental outcomes, Table 6 presents sample detection results, including front and side views of assemblies under various conditions. These images reveal the following observations: Both YOLOv7 and the proposed method accurately detected objects, while YOLOv8s and BGF-YOLO exhibited issues with duplicate detections. Other methods misclassified some irrelevant objects as assemblies, resulting in poorer detection performance. Combined with the data in Table 5, the proposed method’s detection speed surpasses YOLOv7, further emphasizing its advantages in practical applications. Overall, the proposed method outperforms other methods in terms of comprehensive detection performance.

### 4.4. Validation Experiments for Dynamic Object Detection

**Figure 9:**
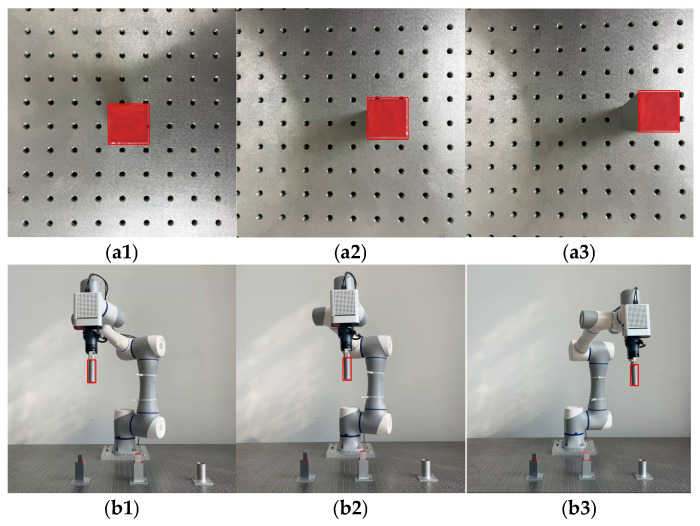
Validation results for dynamic object detection experiments. (**a1**–**a3**) The test results of detecting assembly components using the end camera; (**b1**–**b3**) The test results of robotic arm gripping components during dynamic movement.

To evaluate the effectiveness of the proposed method in identifying dynamic assembly objects, sequential frames from long video sequences were used as the test set. Representative images were selected to demonstrate the performance. Figure 9 presents the experimental results, which include detections from an end-effector camera targeting assemblies on the platform and a third-person camera tracking the robotic arm grasping assemblies. Figure 9(a1–a3) depict the test results of detecting assembly components using the end camera, demonstrating the object recognition capability of the components under different positions on the platform and in the presence of shadows during movement. Meanwhile, b1, b2, and b3 illustrate the test results of the robotic arm gripping components during dynamic movement, highlighting the reliability of the proposed method in dynamic environments.

**Figure 10:**
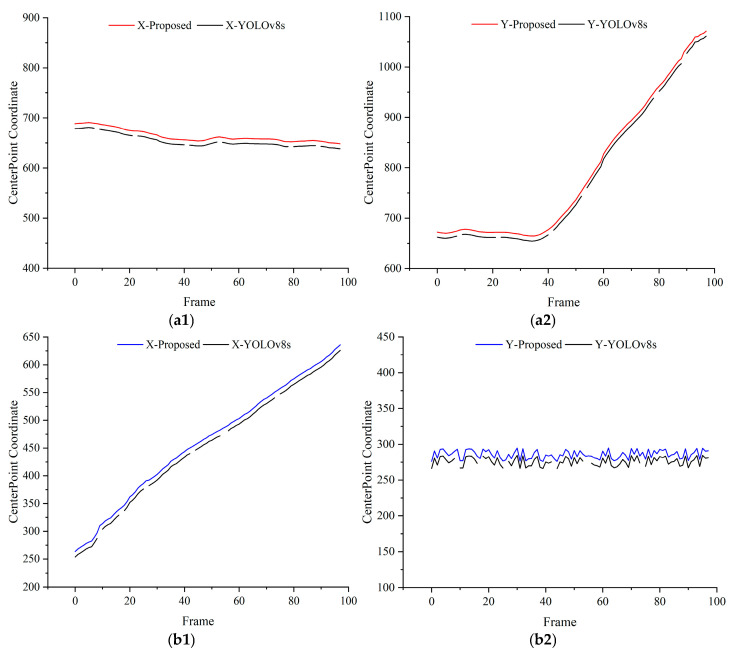
Detection results at 50% speed for dynamic object tracking. (**a1**,**a2**) Comparisons of X and Y coordinates between YOLOv8s and the proposed method using the end camera. (**b1**,**b2**) Comparisons of X and Y coordinates between YOLOv8s and the proposed method using the third-perspective camera. For clarity, the center point of the proposed method’s detection curve is intentionally shifted by 10 units along the *Y*-axis.

**Figure 11:**
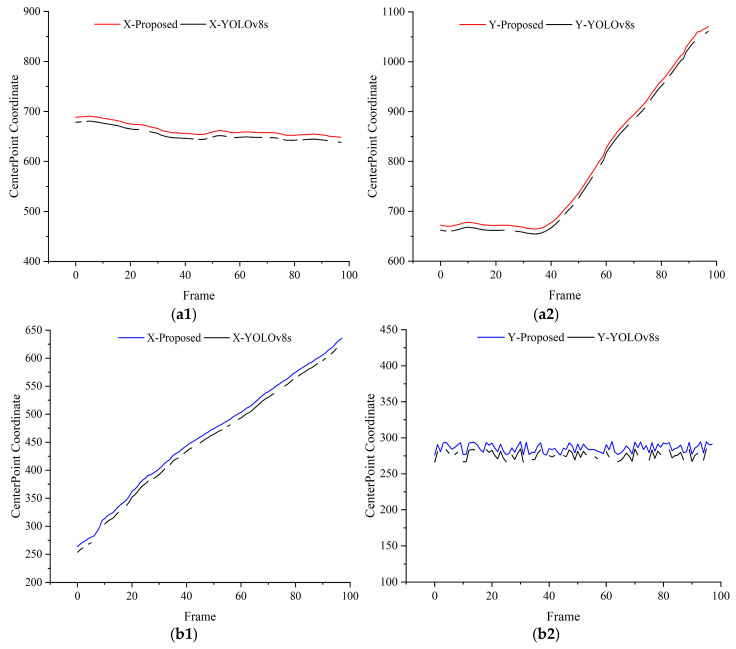
Detection results at 80% speed for dynamic object tracking. (**a1**,**a2**) Comparisons of X and Y coordinates between YOLOv8s and the proposed method using the end camera. (**b1**,**b2**) Comparisons of X and Y coordinates between YOLOv8s and the proposed method using the third-perspective camera. For clarity, the center point of the proposed method’s detection curve is intentionally shifted by 10 units along the *Y*-axis.

**Figure 12:**
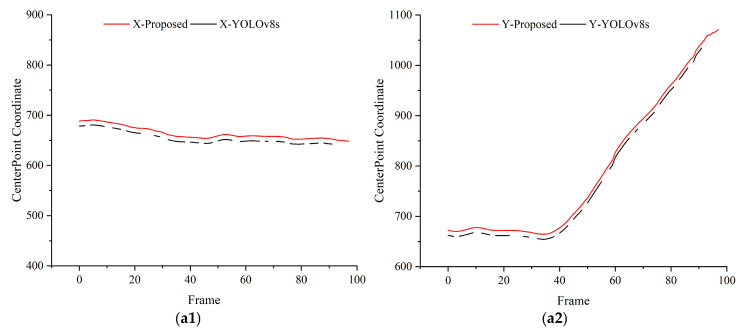

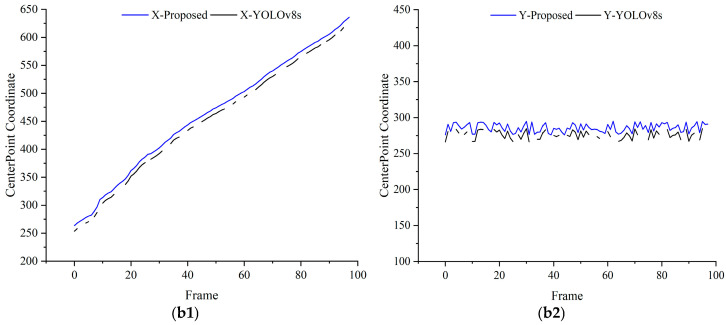
Detection results at 100% speed for dynamic object tracking. (**a1**,**a2**) Comparisons of X and Y coordinates between YOLOv8s and the proposed method using the end camera. (**b1**,**b2**) Comparisons of X and Y coordinates between YOLOv8s and the proposed method using the third-perspective camera. For clarity, the center point of the proposed method’s detection curve is intentionally shifted by 10 units along the *Y*-axis.

To verify the reliability of the proposed method in dynamic recognition of objects in assembly processes, the center points of detection bounding boxes were extracted, and their pixel coordinates were plotted as curves. The continuity of these curves was analyzed to assess detection stability. Taking the detection of assembly components by the end-camera detection platform and the gripping of components by the robotic arm from a third-person perspective as examples, detection curves were drawn as shown in Figure 10, Figure 11 and Figure 12. X and Y represent the horizontal and vertical coordinates of the pixel center points, respectively. Specifically, Figure 10 illustrates the detection results from the end camera and third-perspective camera when the robotic arm operates at 50% speed. Similarly, Figure 11 and Figure 12 present the detection results at 80% and 100% speeds, respectively. In all figures, (a) represents the detection results obtained from the end camera, while (b) represents the detection results obtained from the third-perspective camera.

The results reveal that the YOLOv8s detection curves exhibit obvious discontinuities, indicating instability in dynamic environments. In contrast, the proposed method produces smooth and continuous curves without abrupt peaks, demonstrating improved reliability in identifying dynamic assembly objects.

## 5. Conclusions

This study proposed an improved method for dynamic object detection based on the YOLOv8s architecture, integrating Focused Linear Attention, GhostNet, and VariFocal Loss to enhance the model’s performance in dynamic object detection tasks. The theoretical foundations were detailed, and a series of validation experiments were conducted. Ablation experiments result in the effectiveness of each module. Experiments on the custom dataset have proven the effectiveness of the proposed method, with a Precision of 93.0% and a mAP@0.5 of 92.6%, surpassing the original model and six other object detection methods. Moreover, the proposed method maintains good detection speed, achieving an FPS of 166.9, and has a low parameter count of 9.4 M. Experiments on the MS-COCO dataset further validate the method’s applicability, with a mAP@0.5 of 62.2%. Dynamic object detection experiments showcased its potential for advanced robotic tasks, laying the groundwork for real-time skill imitation. This study lays the foundation for integrating detection algorithms with skill learning systems and provides valuable references for robotics, artificial intelligence, and real-time monitoring. The next step is to further optimize the model and deploy it on a laboratory robot control platform.

## Figures and Tables

**Table 1:** Composition of the custom dataset.

Sample Category	Training	Validation	Test	Total
Front of the cylindrical	600	129	129	858
Side of the cylindrical	1056	226	226	1508
Front of the cubic	602	129	129	860
Side of the cubic	1136	243	243	1622
Front of the triangular prism	597	128	127	852
Side of the triangular prism	1074	230	230	1534

**Table 2:** Software configuration for the experimental setup.

Name	Specific Information
processor	AMD EPYC 9654
GPU	NVIDIA GTX4060 (NVIDIA Corporation, Santa Clara, CA, USA)
Cuda	11.8
cuDNN	8.7.0
Python3	3.7
PyTorch	2.1.0

**Table 3:** Results of ablation experiments. The "√" symbol indicates the presence or utilization of the respective module in the model configuration.

No.	C2f-G	C2f-F	VFL	mAP@0.5	GFLOPs	FPS	Inference Time	Parameters (Million)
1				88.5	28.4	154.1	6.5	11.1
2	√			86.8	24.4	173.8	5.7	8.3
3		√		91.7	27.6	157.2	6.3	10.8
4			√	90.6	28.4	154.3	6.5	11.1
5	√	√		91.1	24.9	167.0	5.9	9.4
6		√	√	92.9	27.7	157.2	6.3	10.8
7	√	√	√	92.6	24.6	166.9	5.9	9.4

**Table 4:** Comparative results of object detection on the public dataset.

Network	mAP@0.5–0.95	mAP@0.5	GFLOPs	FPS	Inference Time	Parameters (Million)
EfficientDet	38.3	40.2	6.6	107.8	9.3	6.1
DETR	42	62.4	86.0	34.1	29.3	41.0
YOLOv7	55.2	69.7	104.7	161.4	6.2	36.9
YOLOv7-tiny	37.2	56.0	13.8	286.0	3.5	6.2
YOLOv8s	44.6	61.2	28.4	173.3	5.8	11.2
BGF-YOLO	43.2	60.5	17.4	178.4	5.6	22.0
Proposed	45.3	62.2	24.6	183.6	5.4	9.4

**Table 5:** Results of comparative experiments on the custom dataset.

Network	Recall	Precision	mAP@0.5–0.95	mAP@0.5	FPS	GFLOPs	Inference Time	Parameters (Million)
EfficientDet	68.4	69.3	67.3	72.1	100.1	6.6	9.9	6.1
DETR	70.8	75.2	72.6	76.2	30.7	86.0	32.5	41.0
YOLOv7	92.9	92.6	76.3	93.3	161.4	104.7	6.2	36.9
YOLOv7-tiny	68.0	75.4	60.2	73.7	286.0	13.8	3.5	6.2
YOLOv8s	89.1	88.7	67.9	88.5	154.1	28.4	6.5	11.2
BGF-YOLO	80.3	85.8	79.4	83.1	106.4	17.4	9.4	22.0
Proposed	91.7	93.0	74.7	92.6	166.9	24.6	5.9	9.4

**Table 6:** Object detection results on the custom dataset.

Network	Front of Assembly (1)	Side of Assembly (1)	Front of Assembly (2)	Side of Assembly (2)
EfficientDet	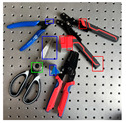	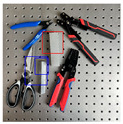	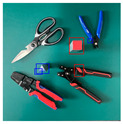	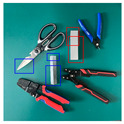
DETR	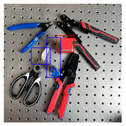	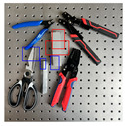	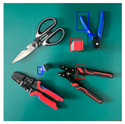	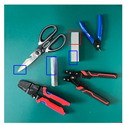
YOLOv7	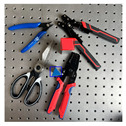	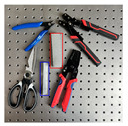	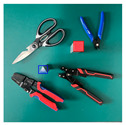	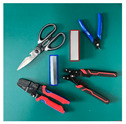
YOLOv7-tiny	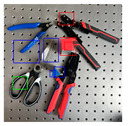	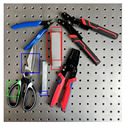	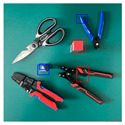	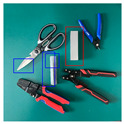
YOLOv8s	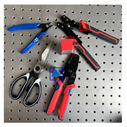	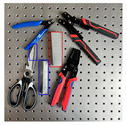	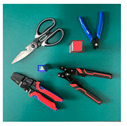	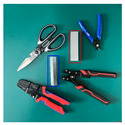
BGF-YOLO	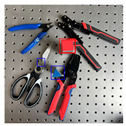	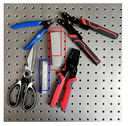	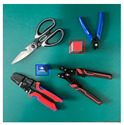	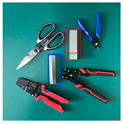
Proposed	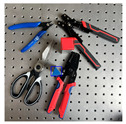	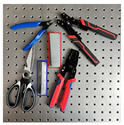	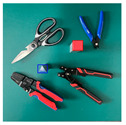	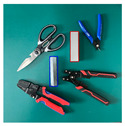

## Data Availability

The original contributions presented in the study are included in the article; further inquiries can be directed to the corresponding author.

## References

[1] Liu H., Liu T., Zhang Z., Sangaiah A.K., Yang B., Li Y. (2022). ARHPE: Asymmetric Relation-Aware Representation Learning for Head Pose Estimation in Industrial Human-Computer Interaction. IEEE Trans. Ind. Inf..

[2] Dos Reis D.H., Welfer D., Cuadros M.A., Gamarra D.F. (2020). Object Recognition Software Using RGBD Kinect Images and the YOLO Algorithm for Mobile Robot Navigation. Intelligent Systems Design and Applications.

[3] Wang C., Moqurrab S.A., Yoo J. (2023). Face Recognition of Remote Teaching Video Image Based on Improved Frame Difference Method. Mobile Netw. Appl..

[4] Liu Y., Fu Y., Zhuan Y., He X. (2021). High Dynamic Range Real-Time 3D Measurement Based on Fourier Transform Profilometry. Opt. Laser Technol..

[5] Hu M., Chen Y., Hu H., He Z. (2024). Single Frame Digital Phase-Shift Fringe Projection Profilometry Based on Symmetry Transform. Opt. Eng..

[6] Zhong M., Hu Z., Duan P., Hu X. (2024). Modulation Measurement Profilometry Based on One-Dimensional Frequency-Guided S-Transform. J. Phys. Conf. Ser..

[7] Dai M., Peng K., Luo M., Huang Y. (2020). Dynamic Phase Measuring Profilometry for Rigid Objects Based on Simulated Annealing. Appl. Opt..

[8] Wang Y., Abd Rahman A.H., Nor Rashid F.’A., Razali M.K.M. (2024). Tackling Heterogeneous Light Detection and Ranging-Camera Alignment Challenges in Dynamic Environments: A Review for Object Detection. Sensors.

[9] Tan Q., Du Z., Chen S. (2023). Moving Target Detection Based on Background Modeling and Frame Difference. Procedia Comput. Sci..

[10] Alfarano A., Maiano L., Papa L., Amerini I. (2024). Estimating Optical Flow: A Comprehensive Review of the State of the Art. Comput. Vis. Image Underst..

[11] Gude G.N.M.R., Karthikeyan P.R. (2023). Frame Differencing, a Single Gaussian, and Modified GMM for Foreground Object Detection on Camera Jitter Movies in Comparison to F-Score Measurement. J. Surv. Fish. Sci..

[12] Saxena S., Herrmann C., Hur J., Kar A., Norouzi M., Sun D., Fleet D.J. (2024). The Surprising Effectiveness of Diffusion Models for Optical Flow and Monocular Depth Estimation. Adv. Neural Inf. Process. Syst..

[13] Liu S., Wang Y., Yu Q., Liu Y. (2023). A Driver Fatigue Detection Algorithm Based on Dynamic Tracking of Small Facial Targets Using YOLOv7. IEICE Trans. Inf. Syst..

[14] Cao Z., Liao T., Song W., Yang F. (2021). Detecting the Shuttlecock for a Badminton Robot: A YOLO-Based Approach. Expert Syst. Appl..

[15] An Y., Li Z., Li Y., Zhang K., Zhu Z., Chai Y. (2024). Few-Shot Learning-Based Fault Diagnosis Using Prototypical Contrastive-Based Domain Adaptation under Variable Working Conditions. IEEE Sens. J..

[16] Li J., Wei R., Zhang Q., Shi R., Jiang B. (2024). Research on Real-Time Roundup and Dynamic Allocation Methods for Multi-Dynamic Target Unmanned Aerial Vehicles. Sensors.

[17] Schmid L., Andersson O., Sulser A., Pfreundschuh P., Siegwart R. (2023). Dynablox: Real-Time Detection of Diverse Dynamic Objects in Complex Environments. IEEE Robot. Autom. Lett..

[18] Jocher G., Chaurasia A., Qiu J. (2023). Ultralytics YOLO.

[19] Yin Q., Lu W., Li B., Huang J. (2023). Dynamic Difference Learning with Spatio–Temporal Correlation for Deepfake Video Detection. IEEE Trans. Inf. Forensics Secur..

[20] Delibaşoğlu İ. (2023). Moving Object Detection Method with Motion Regions Tracking in Background Subtraction. Signal Image Video Process..

[21] Zhang Q.L., Li S.L., Duan J.G., Liu R., Hu J. (2024). Moving Object Detection Method Based on the Fusion of Online Moving Window Robust Principal Component Analysis and Frame Difference Method. Neural Process. Lett..

[22] Yang B., Xie H., Li H., Liu Q. (2020). Unsupervised Optical Flow Estimation Based on Improved Feature Pyramid. Neural Process. Lett..

[23] Hu B., Luo J., Gao J., Fan T., Zhao J. (2023). A Robust Semi-Direct 3D SLAM for Mobile Robots Based on Dense Optical Flow in Dynamic Scenes. Biomimetics.

[24] Ding J., Zhang Z., Yu X., Zhao X., Yan Z. (2023). A Novel Moving Object Detection Algorithm Based on Robust Image Feature Threshold Segmentation with Improved Optical Flow Estimation. Appl. Sci..

[25] Tan M., Pang R., Le Q.V. EfficientDet: Scalable and Efficient Object Detection. Proceedings of the IEEE/CVF Conference on Computer Vision and Pattern Recognition (CVPR).

[26] Carion N., Massa F., Synnaeve G., Usunier N., Kirillov A., Zagoruyko S. (2020). End-to-End Object Detection with Transformers. Proceedings of the European Conference on Computer Vision (ECCV).

[27] Redmon J., Divvala S., Girshick R., Farhadi A. You Only Look Once: Unified, Real-Time Object Detection. Proceedings of the IEEE Conference on Computer Vision and Pattern Recognition (CVPR).

[28] Wang C.Y., Bochkovskiy A., Liao H.Y.M. YOLOv7: Trainable Bag-of-Freebies Sets New State-of-the-Art for Real-Time Object Detectors. Proceedings of the IEEE/CVF Conference on Computer Vision and Pattern Recognition (CVPR).

[29] Kang M., Ting C.M., Ting F.F., Chen L., Zhang R., Ma Y. (2024). BGF-YOLO: Enhanced YOLOv8 with Multiscale Attentional Feature Fusion for Brain Tumor Detection. Proceedings of the International Conference on Medical Image Computing and Computer-Assisted Intervention (MICCAI).

[30] An Q., Chen X., Zhang J., Shi R., Yang Y., Huang W. (2022). A Robust Fire Detection Model via Convolution Neural Networks for Intelligent Robot Vision Sensing. Sensors.

[31] Zhang X., Fu Q., Li Y., Wang Z. (2024). A Dynamic Detection Method for Railway Slope Falling Rocks Based on the Gaussian Mixture Model Segmentation Algorithm. Appl. Sci..

[32] Zhao L., Qiu S., Chen Y. (2024). Enhanced Water Surface Object Detection with Dynamic Task-Aligned Sample Assignment and Attention Mechanisms. Sensors.

